# Epigenetic changes induced by parasitic worms and their excretory-secretory products

**DOI:** 10.1042/BST20230087

**Published:** 2024-02-09

**Authors:** William Harnett, Margaret M. Harnett

**Affiliations:** 1 Strathclyde Institute of Pharmacy and Biomedical Sciences, University of Strathclyde, Glasgow, U.K.; 2 School of Infection and Immunity, University of Glasgow, Glasgow, U.K.

**Keywords:** E–S products, epigenetics, immunomodulation, inflammation, parasitology

## Abstract

Parasitic worms are pathogens of major medical and veterinary importance. They have evolved highly effective and sophisticated strategies of immune system manipulation, typically involving actively excreted/secreted (E–S) products. These molecules dampen and regulate the host immune responses that would otherwise result in parasite expulsion, thereby enabling the worms to survive in the host for many years, and they can also help prevent the potentially serious tissue damage that the worms can induce. Reflecting these E–S product-associated anti-inflammatory activities, there is also increasing evidence that parasitic worms and their products may serendipitously protect against allergic and autoimmune conditions and in addition, comorbidities of ageing that are associated with inflammatory responses, like type 2 diabetes and obesity. Research in this area has to date generally focused on identifying the cellular and effector targets of immunomodulation induced by the worm E–S products. However, increasing evidence that they can induce stably imprinted phenotypes of haematopoietic and stromal cells which promote their long-lasting survival has recently ignited interest in the ability of the molecules to epigenetically rewire cells to ‘resolve and repair’ phenotypes. Here, we review and discuss these new data in the context of their potential for exploitation in identifying novel gene signatures for the development of advanced and safe therapeutics for chronic inflammatory diseases.

## Induction and persistence of a distinct immunological phenotype following parasitic worm infection

Worms are highly successful parasites of both invertebrates and vertebrates and it has been estimated that species transmitted via soil alone may infect in the region of one quarter of the human population [1]. A common feature of their parasitism is that it is very long lasting, for example species of filarial nematode, which cause elephantiasis in humans may survive for at least a decade [2]. This dictates that the worms must have in place, very effective strategies for evading the multiple components of the host immune system. Certainly, the worms are generally not invisible to the host's immune system and regardless of worm species they are quickly sensed and a response mounted by stromal cells like keratinocytes and epithelial cells, which release alarmin molecules including IL-25, IL-33 and thymic stromal lymphopoietin (reviewed in [3]). These act on type 2 innate lymphoid cells, which secrete cytokines such as IL-5 and IL-13, and the latter in particular can act on dendritic cells (DCs) to help them polarise naïve T cells towards a T helper 2 (Th2) phenotype (reviewed in [4]). Moreover, worm-derived molecules may be taken up by DCs, also impacting these cells with properties that drives Th2 polarisation [5]. A Th2 response is in fact a general feature of parasitic worm infection but as it can lead to worm elimination and/or severe host pathology it is often modified by the induction of an anti-inflammatory component as shown by the appearance of cytokines such as IL-10 and TGF-β and cell-types like alternatively activated macrophages and regulatory B and T cells (reviewed in [6]). The induction of this anti-inflammatory immunologic phenotype is considered to be a strong contributor to parasitic worm longevity.

It is generally assumed that induction of the Th2/anti-inflammatory phenotype that persists during parasitic worm infection reflects interaction of the host immune system with molecules that are actively excreted or secreted (excretory-secretory products; E–S products) by the worms and indeed many worm-derived molecules have now been shown to drive Th2 or regulatory cell responses (reviewed in [7,8]). Consistent with this, a series of studies have reported that elimination of worm infection, and hence termination of E–S product production, via chemotherapy, can result in reversal of some features of the phenotype (reviewed in [9]). The mechanisms by which E–S impact on the immunological phenotype of the host have been subject to considerable investigation over the past few decades and a great deal of information has been accumulated (reviewed in [6–10]). One idea which has recently begun to be explored by looking, for example, at changes in DNA methylation or histone modifications, is that worm E–S products may epigenetically alter immune system cells. Such changes can occur in cells of both the innate and adaptive immune response and are often referred to as a form of ‘training’ in that they can result in an enhanced response by the cell when exposed to the inducing stimulus on a second occasion (reviewed in [11,12]). The idea that parasitic worm E–S products can cause epigenetic changes in cells will be considered in this mini-review but first, the focus will be on studies using whole living worms *per se*.

## Evidence from parasitic worm infection in mice

An idea which has received substantial investigation for several decades is that the changes in immunological phenotype, which persist during parasitic worm infection can protect humans from developing autoimmune and allergic conditions. One condition that has been particularly well explored is asthma, and some of the studies undertaken have indeed generated evidence consistent with parasitic worms offering protection (reviewed in [13]). Even more convincing are the data arising from mouse model studies, where a range of different parasitic worm species have been shown to protect against allergic airway hyper-responsiveness in the lungs (reviewed in [7]). Associated with these studies, there has been interest in whether worm infection during pregnancy might modify the susceptibility of the new-born to develop asthma as they age. This has been explored in a model employing pregnant mice infected with the medically important trematode parasitic worm of humans, *Schistosoma mansoni*. Using this model, it was discovered that if female mice mated during the period of infection when they were subject to immunosuppression/immunoregulation by the worms, then their offspring were found to be protected against developing ovalbumin-induced airway hypersensitivity [14]. Subsequent analysis of naïve T cells from the infected offspring revealed that their capacity to differentiate in a Th2 direction was impaired as measured by production of IL-4 and this was correlated with significantly decreased expression of the key transcription factor necessary for Th2 progression, GATA-3 [15]. Furthermore, in support of a mechanism of action dependent on epigenetic modification, reduced histone acetylation of the IL-4 promoter region was noted.

In addition to inducing epigenetic changes in lymphocytes, *S. mansoni* can also generate epigenetic alterations in innate immune system cells like the macrophage. The worm infection typically polarises macrophages in an M2 direction, a Th2/IL-4-driven phenotype associated with tissue-repair rather than anti-microbial activities [16]. Studies using an *S. mansoni* egg challenge mouse model revealed that M2 macrophage polarisation in mice exposed to the eggs was dependent on reduced H3K27me2/3 levels at specific M2 marker gene promoters and increased global expression of Jmjd3, the methylase that removes the marks [17]. Of note, the latter increase was enhanced by earlier exposure to *S. mansoni*. Furthermore, schistosome eggs also impact on the capacity of DCs to drive Th2 responses, and that this has been reported as being dependent on the methyl-CpG-binding protein, Mbd2, which has a role in coordinating chromatin accessibility and hence reprogramming of gene expression in response to DNA methylation, is further evidence for schistosome parasitic worms impacting epigenetic changes in immune system cells [18].

## Evidence from parasitic worm infection in humans

Investigation of epigenetic changes induced by schistosome parasites (the related *Schistosoma haematobium*) and also the gastrointestinal nematode, *Ascaris lumbricoides* (considered the parasitic worm most commonly infecting humans) has also been undertaken employing primary immune system cells from children recently exposed to *Mycobacterium tuberculosis*. When infected with the worm parasites, the children revealed 751 differentially DNA methylated genes of which 72% were hypermethylated [19]. These changes were associated with inhibition of pathways necessary for an effective immune response to *M. tuberculosis* including IFN-γ signalling, cellular proliferation and Th1 polarisation and thus offer a molecular explanation for the increased tuberculosis disease progression observed in parasitic worm infected individuals [20]. In the individuals infected with *S. haematobium*, the changes in methylation persisted for at least 6 months after deworming via chemotherapy [19].

## Evidence from extracts and E–S products of *Fasciola hepatica*

Studies employing the trematode parasite *Fasciola hepatica* revealed that exposure of mouse macrophages to a soluble total worm extract of the adult life cycle stage (FHTE) resulted in production of the interleukin-1 receptor antagonist protein, IL-1RA, and the anti-inflammatory cytokine IL-10 and also inhibition of production of the pro-inflammatory cytokine, TNF, in response to bacterial lipopolysaccharide (LPS) [21]. The extract thus shows one of the classic characteristics of worm parasites in inducing an anti-inflammatory immune phenotype. Interestingly however, if the cells were pre-exposed to the FHTE, allowed to recover and then re-exposed to FHTE, higher levels of IL-1RA and IL-10 were induced [21]. Similarly, pre-treatment with FHTE and then exposure to LPS had the same effect in increasing IL-1RA and IL-10 levels but reducing TNF levels. These data are consistent with the idea of FHTE training macrophages to increase their anti-inflammatory response and the use of the methyltransferase inhibitor MTA to successfully inhibit the effects [21] suggested a role for histone methylation and hence epigenetic modification in their generation. A similar anti-inflammatory macrophage phenotype could be generated *in vivo* by inoculating mice with FHTE and recovery of these cells and their transfer into mice undergoing experimental allergic encephalomyelitis (EAE), a model of multiple sclerosis, delayed the clinical course and weight loss associated with the disease [21].

Moving on to *F. hepatica* E–S products, it has been shown that the use of this molecular fraction obtained from adult worms (FHES) can also impact on the phenotype of mouse immune system cells. Thus, macrophages prepared from bone marrow (BM) recovered from mice exposed to FHES secrete increased levels of IL-1RA and IL-10 but decreased levels of the pro-inflammatory cytokine, IL-1β, following LPS stimulation [22]. Impressively, some of these effects were found to persist for 18 months. Furthermore, as with FHTE, FHES-treated macrophages were found to transfer protection against features of EAE and in these experiments, there was still a degree of protection eight months after FHES exposure [22].

Further investigative work showed that administered FHES reached the mouse BM revealing a potential to interact with BM cell populations and this was confirmed for example by the observation that mice treated with FHES had increased numbers of long-term haematopoietic stem cells (LT-HSCs) and a noteworthy rise in the common monocyte precursor population [22]. Moreover, transfer of whole BM cells from FHES-treated mice into recipient mice resulted in the generation of a peritoneal or BM-derived macrophage population harbouring the anti-inflammatory cytokine secretion profile observed in the earlier *in vitro* work and this population was also found to provide protection against EAE. In addition, such protection was also obtained when transferring LT-HSC from FHES-exposed animals [22]. Subsequent work using the pan-histone deacetylase inhibitor givinostat showed it to impede increased IL-6 (a cytokine which can have anti-inflammatory activity), but not IL-10, production by BM cells obtained from naïve mice and exposed to FHES followed by CpG oligodeoxynucleotides (drive inflammatory responses via TLR9) *in vitro* [22]. However, inhibition of mTOR by rapamycin inhibited the production of IL-10 and it was thus concluded that overall the changes induced by FHES reflected both epigenetic modifications and altered metabolic activity.

## Evidence from a single defined parasitic worm E–S product, ES-62

**Figure 1.**
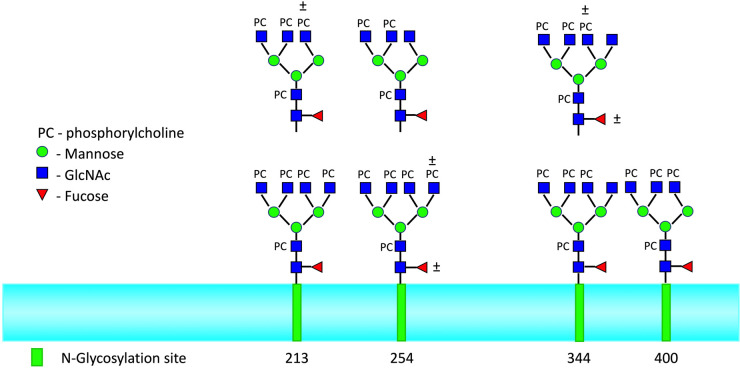
Location and structure of immunomodulatory phosphorylcholine (PC)-*N*-glycans of ES-62. ES-62, a secreted protein of *A. viteae* has immunomodulatory properties due to the presence of PC on multiple *N*-glycans attached to the protein backbone. The diagram shows different PC-*N*-glycan forms which have been detected at the protein's 4 *N*-glycosylation sites, with their amino acid position shown [24]. The protein exists as tetramer in its native form with each tetramer having the potential to express up to 72 PC groups, which potentially can interact with molecules and cells of the immune system. GlcNAc represents *N*-acetylglucosamine.

ES-62 is the major E–S product of the rodent filarial nematode *Acanthocheilonema viteae* [23]. The molecule is a tetrameric protein that contains multiple phosphorylcholine moieties attached to *N*-type glycans [24], which provide it with a range of immunomodulatory properties (Figure 1 and reviewed in [25]). For example, *in vitro* exposure of antigen presenting cells such as BM-derived macrophages [26] or DCs [27] to ES-62 interferes with their ability to secrete cytokines IL-12 (can be pro-inflammatory, driving autoimmune conditions) in response to LPS. Interestingly, if macrophages or DCs are prepared *ex vivo* from BM obtained from mice exposed to ES-62 via osmotic pumps, these cells demonstrate similarly impaired IL-12 cytokine production in response to LPS exposure [26]. Maturation of macrophages and DCs *in vitro* takes ∼1 week and so these results would appear to indicate that exposure to ES-62 can hardwire BM progenitor cells such that following maturation they cannot respond normally to an inflammation-inducing agent like LPS. Furthermore, support for the idea that ES-62 can stably retrain BM cells to an anti-inflammatory phenotype is provided by our studies showing that *ex vivo* derived BM-DCs displayed a reduced ability to prime (pathogenic) Th17 cytokine responses when exposed to ES-62 *in vivo* in a mouse model of asthma [28]. As far as we are aware these data represent the first example of a defined molecule secreted by a pathogen subverting the maturation of innate immune system cell progenitors in the BM and as alluded to above, are consistent with the idea of ES-62 inducing epigenetic modifications. The ability of ES-62 to epigenetically modify immune system cells will almost certainly impact on their functional significance during infection with *A. viteae*. Furthermore, ES-62's plethora of immunomodulatory activities dictate that it is protective in many models of allergy and autoimmunity [7,24], with one of the latter being the MRL/lpr mouse strain used in the study of systemic lupus erythematosus. In this model, prophylactic administration of ES-62 is very effective at preventing mice from developing kidney damage [29]. Mechanistically, this protection is associated with a restoration of the imbalance which develops between effector and regulatory B cells and which drives disease via auto-antibody production, and strikingly, the protection can be transferred to recipient mice by ‘trained’ splenic B cells from ES-62 exposed mice. Thus, the ability of ES-62 to stably modify immune system cells to an anti-inflammatory phenotype is important with respect to the therapeutic potential of the molecule.

Further support for ES-62 inducing epigenetic changes in cells has arisen from studies on not an immune system cell but rather a stromal cell, the synovial fibroblast (SF). These cells have recently received attention due to increasing awareness of their multiple roles in rheumatoid arthritis pathogenesis (reviewed in [30]). During pathogenesis, SFs assume a mesenchymal/fibrotic phenotype [31], undergoing epigenetic rewiring as a consequence of the hypoxia and inflammation developing in the microenvironment of the arthritic joint [32], to stably display aberrant migratory, proliferative and pro-inflammatory responses. This rewiring is demonstrated by alterations in the SF's global DNA methylation condition, predominantly in the hypomethylation of promoter regions linked to the de-repression of inflammatory genes driving SF transformation [31–33]. Amongst ES-62's anti-inflammatory properties is an ability to protect mice against collagen-induced arthritis (CIA), a model for human rheumatoid arthritis (reviewed in [34]). ES-62 achieves protection via many mechanisms including interfering with the pro-inflammatory response of SFs, e.g. *ex vivo*, the cells produce less IL-6 (pro-inflammatory in this context) either spontaneously or in response to IL-17 than cells from control CIA-mice [35]. To investigate whether this is a sign of stable rewiring of their functional phenotype, SF explant cultures, passaged for 3–4 weeks, were examined for release of pro-inflammatory mediators [36]. It was found that IL-6 secretion arising spontaneously or in response to various arthritogenic stimuli (IL-17, IL-1β, LPS, bacterial lipo-peptide) was significantly reduced in explant cultures from CIA-mice treated with ES-62 relative to control CIA-mice, indeed it declined to a level similar to non-arthritic mice. This result was also observed in the SFs when measuring IL-6 mRNA and also production of the chemokine CCL2, and the matrix metalloproteases, MMP9 and MMP15, at the protein and/or mRNA level. Simultaneously, while the important negative regulators of such pro-inflammatory signalling, SOCS1 and SOCS3, were down-regulated in CIA–SFs, exposure to ES-62 rescued their expression towards the higher levels detected in passaged SFs from non-arthritic animals. Thus, clearly the ability of ES-62 to reverse responses to pro-inflammatory stimuli is maintained in passaged SFs for at least 3–4 weeks, a potential sign of epigenetic modification.

Subsequent mechanistic analysis revealed that CIA–SFs exhibit hypomethylated global DNA and reduced expression of DNA methyltransferase-1 (DNMT1) relative to those from non-arthritic control mice but surprisingly, ES-62 did not simply prevent this. Rather, it was found that ES-62 additionally increased global DNA hypomethylation and further reduced the levels of DNMT1 in SFs from CIA-mice [36]. These data therefore suggest that ES-62 does not simply block transformation of SFs to the pathogenic phenotype during CIA but rather further epigenetically models the cells to a novel ‘resolving’ phenotype. In support of this, subjecting the SFs to reduced Representation Bisulphite Sequencing and bioinformatics analysis revealed each of the three SF groups — CIA, CIA-ES-62, and non-CIA — to possess unique DNA methylation profiles. Furthermore, differential DNA methylation scrutiny of the Promoter and Gene Body zones signposted that ES-62 functioned to preserve the non-arthritic methylation status in only a restricted number of genes and in its place, differentially promoted hypo- and in some cases hyper-DNA methylation of a variety of genes relative to their CIA and non-arthritic counterparts. To probe whether the differential DNA methylation displayed by the three groups could offer insight into the processes responsible for the creation of their functionally distinct SF phenotypes, pathway enrichment analysis using STRING and KEGG databases was performed. This revealed differential promoter silencing of the Ras/MAPkinase, FoxO, Hippo and Wnt signalling pathways that are key to the regulation of many cellular processes subject to dysregulation in SF pathogenesis (with ciliogenesis, a master regulator of signalling networks coordinating cellular and organ development, survival, proliferation and migration, inflammation and tissue repair [38,39], as an interesting example) and by cross-mining these data with genes already implicated in RA pathogenesis, the concept of ES-62 uniquely inducing a protective resolving phenotype was again apparent [36].

The therapeutic potential of ES-62 has been alluded to earlier but as a large, foreign and hence potentially immunogenic protein and whose active moiety is dependent on a post translational event restricted to nematodes and perhaps a few other classes of lower organism, in reality ES-62 is unsuitable for use as a drug. For this reason, a library of PC-based ES-62 Small Molecule Analogues (SMAs) was developed [37,40] and found to contain members which mimicked the therapeutic potential of the parent molecule (reviewed in [25,34]). The ability to epigenetically rewire SFs demonstrated by ES-62 in the CIA model is also shared with these ES-62 SMAs as shown using SMA 12b as an example [36]. Furthermore, adoptive transfer of DCs exposed to a combination of two SMAs, 11a and 12b, during their maturation, protects mice from developing CIA [41]. Thus, the hardwiring in various cell-types that results from exposure to ES-62 can be mirrored by its synthetic drug-like mimetics.

Finally, many parasitic worms are gut dwelling and/or regulate microbiome homeostasis [42,43] and there is increasing evidence that gut microbiome dysbiosis is associated with development and perpetuation of allergic and autoimmune diseases as well as the comorbidities of ageing, like type 2 diabetes and obesity, that are underpinned by chronic inflammation [44,45]. Moreover, it appears that a major factor linking gut dysbiosis to these diseases is the accompanying epigenetic remodelling of haematopoietic and stromal cell differentiation in the BM and the periphery that results in the disruption of haematopoietic homeostasis and consequently, the induction of ‘training’ of pro-inflammatory immune effector phenotypes that drive and perpetuate inflammation [46]. It is therefore intriguing that the protective anti-inflammatory actions of ES-62 against CIA and also, obesity-accelerated ageing in male mice [47–49] are associated with protection of gut integrity and normalisation of the gut microbiota, particularly, in the case of CIA [48], by maintaining/increasing levels of butyrate-producing species as this short chain fatty acid has been associated with the homeostatic epigenetic regulation of the gut-osteoimmunology axis [50–53]. Indeed, in the obesity-accelerated ageing model, ES-62 protects against loss of trabecular bone and acts to maintain the BM niche, resulting in the normalisation of both the adipocyte (stromal) and the myeloid/lymphoid (haematopoietic) biases otherwise associated with such ageing [47].

## Perspectives

*Importance of field*

- Parasitic worms are exceedingly prevalent and medically important pathogens that have evolved highly successful strategies to limit host immune responses to promote their long-term survival. A serendipitous side effect of such immunomodulation is that worm infection appears to afford protection against allergic and autoimmune conditions, as well as ageing-associated comorbidities underpinned by chronic inflammation a proposal supported by the dramatic rise in these diseases accompanying the rapid elimination of these parasites in developed countries over the last century.

*Summary of current thinking*

**Figure 2.**
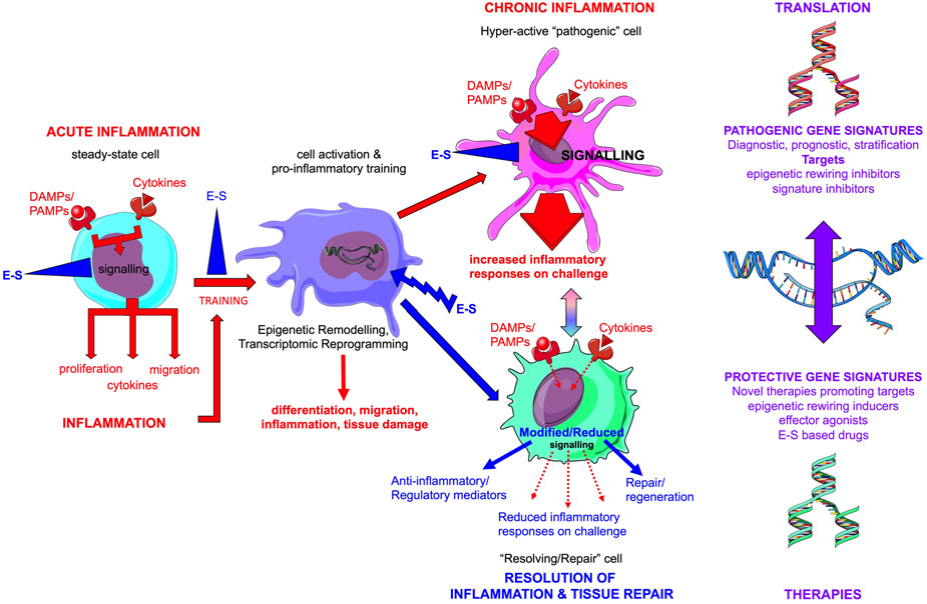
Parasitic worm E–S products epigenetically rewire cells to resolving and repair phenotypes. In acute inflammation, healthy cells (steady-state cells) respond to DAMPs (danger-associated molecular patterns like alarmins)/PAMPs (pathogen-associated molecular patterns like LPS), cytokines and inflammatory mediators resulting in the amplification of inflammation by the consequent tissue infiltration by innate and adaptive immune system cells. Whilst important for combatting infection, failure to resolve such inflammation results in chronic inflammation and development of comorbidities associated with ageing. In addition to perpetuating tissue damage, chronic inflammation drives epigenetic remodelling and transcriptomic reprogramming of haematopoietic and stromal cells in the BM and periphery. Such ‘rewired’ cells are hyper-responsive to environmental cues and play key roles in the development of chronic inflammatory conditions by disruption of immunoregulatory and immunometabolic networks, particularly those associated with the gut-BM axis (all pathogenic signals are denoted by red arrows with imprinted hyper-responsiveness represented by increased arrow size and redness of the ‘inflamed’ cell). E–S products can disrupt this process at multiple points. Firstly, they can suppress (blue blocking symbol) the effects of acute inflammation by targeting downstream signalling pathways and hence limit the recruitment and activation of tissue-infiltrating cells. In addition, they can suppress the signalling generated by chronic inflammation, required to drive epigenetic remodelling of cells to hyper-responsive pathogenic phenotypes, as well as targeting signalling occurring within any rewired cells present during established disease. Critically, E–S products like ES-62 do not appear to simply block pathogenic rewiring of cells but rather, they can induce additional resolving/repair actions (blue lightning bolt symbol) by modulation of signal transduction and further epigenetic remodelling. Moreover, such ‘resolving/repair’ cells may additionally act to modulate the behaviour of the rewired ‘pathogenic’ phenotypes (as indicated by the green/pink gradient arrow). The translational potential in identifying pathogenic and resolving/repair epigenetic gene signatures is indicated, as well as how these may be therapeutically targeted. This model was created, in part, using colour-modified images from Servier Medical Art, provided by Servier under a Creative Commons Attribution 3.0 unported license (https://creativecommons.org/licenses/by/3.0/).

- Identifying the cellular and molecular targets of immune system manipulation by the parasitic worms and their secreted immunomodulatory E–S products has been a major focus, but recent studies have highlighted that the worms can stably ‘rewire’ immune responses by inducing epigenetic remodelling of haematopoietic and stromal cells and/or their progenitors and that such ‘training’ can be inherited. The challenge now is to identify the ‘resolve and repair’ gene signatures induced by the parasitic worms in various disease settings (Figure 2).

*Future directions*

- Recognition that the actions of the parasitic worms can be recapitulated by defined E–S products has made exploitation of their therapeutic potential a reality and indeed, development of E–S SMAs offers the possibility of safe, cheap and easily manufactured drugs. That such SMAs can be potentially designed to exhibit different aspects of E–S action offers the possibility of ‘personalised’ drug cocktails following patient stratification.
- Finally, since E–S products do not simply act to prevent pathogenic immune responses but can direct further cell differentiation to novel ‘protective/repair’ phenotypes that resolve inflammation and prevent tissue destruction, the associated epigenetic gene signatures may signpost fresh therapeutic targets not only for chronic inflammatory conditions but also for regenerative medicine.

## Abbreviations

BM: bone marrow
CIA: collagen-induced arthritis
DC: dendritic cell
DNMT1: DNA methyltransferase-1
EAE: experimental allergic encephalomyelitis
LPS: lipopolysaccharide
LT-HSC: long-term haematopoietic stem cell
SF: synovial fibroblast
SMA: Small Molecule Analogue
